# Evolution of Approaches to the Development, Life Cycle Control, and Interchangeability of Veterinary Biosimilars Based on Hemoproteins (with a Focus on Cytochrome C)

**DOI:** 10.3390/ph19010063

**Published:** 2025-12-29

**Authors:** Vladimir S. Ponamarev

**Affiliations:** Department of the Pharmacology and Toxicology, Saint-Petersburg State University of Veterinary Medicine, Chernigovskaya St., 196084 St. Petersburg, Russia; nir@spbguvm.ru or psedopyos@mail.ru

**Keywords:** pharmacology, biosimilars, veterinary medicine, Cytochrome C

## Abstract

**Background/Objectives:** Biosimilars are central to the modernization of veterinary pharmacology, improving access to complex biological therapies while maintaining quality, safety, and efficacy. Hemoproteins such as cytochrome c, used to support liver function and manage metabolic disorders in animals, are of particular interest. However, their structural complexity and species-specific pharmacology create significant analytical and regulatory challenges for biosimilar development and life-cycle management. Addressing these issues is critical for improving therapeutic outcomes and enabling the broader adoption of biosimilars in veterinary practice. **Methods:** This narrative review examines the scientific and regulatory principles underlying the development of veterinary biosimilars of hemoproteins, with cytochrome c as a representative model. Regulatory guidelines and relevant scientific literature were analyzed to identify key challenges, knowledge gaps, and required adaptations from human to veterinary medicine, with a focus on biosimilar assessment and life-cycle management. **Results:** Veterinary biosimilar frameworks are largely informed by EU and US regulatory pathways, emphasizing the stepwise demonstration of biosimilarity through extensive analytical and functional characterization. Long-term safety and efficacy depend on robust Pharmaceutical Quality Systems and effective life-cycle management to ensure manufacturing consistency. For cytochrome c, interchangeability may be acceptable when analytical similarity is exceptionally high. Critical Quality Attributes include polypeptide integrity, heme–protein interaction, iron redox state, and correct three-dimensional conformation. Quality by Design approaches are essential to control manufacturing variability. Despite regional regulatory differences, core scientific principles remain consistent. **Conclusions:** Hemoprotein biosimilars hold significant promise in veterinary medicine, provided their development is supported by rigorous analytical characterization, strong life-cycle management, and science-based regulatory approaches.

## 1. Introduction

**The Importance of Biosimilars in Modern Veterinary Pharmacology**

The landscape of veterinary medicine is undergoing a profound transformation, increasingly adopting advanced therapeutic modalities previously reserved for human healthcare. This shift is driven by a growing understanding of complex pathophysiological mechanisms in animals, the rising importance of companion animals as family members, and the economic necessity of maintaining the health of livestock. In this context, biopharmaceuticals, or biological medicinal products, have emerged as crucial tools [1,2,3]. These large, complex molecules, produced from living organisms, offer targeted therapeutic actions for a range of conditions, from autoimmune diseases to cancer and metabolic disorders. However, the high cost of innovator biologicals often limits their accessibility in veterinary practice.

Biosimilars, which are biological products highly similar to an already approved reference biopharmaceutical, represent a pivotal solution to this challenge. They foster market competition, reduce healthcare costs, and improve patient access to cutting-edge therapies. The development and approval of biosimilars are founded on the principle that once the patent for an innovator biological expires, other manufacturers can develop versions that demonstrate comparable quality, safety, and efficacy. The regulatory pathway for biosimilars, well-established in human medicine in regions like the European Union and the United States since the mid-2000s [4,5], is now gaining traction in the veterinary field, promising a new era of affordable, high-quality biological treatments for animals.

The scientific and regulatory paradigm for biosimilars is distinct from that of generic small-molecule drugs. Due to the inherent complexity and variability of biological molecules, a biosimilar is not an exact generic copy. Instead, its approval relies on a comprehensive comparability exercise that demonstrates a high degree of similarity to the reference product through a rigorous head-to-head comparison [6,7]. This approach avoids the unnecessary repetition of non-clinical and clinical studies, streamlining development while ensuring patient safety [8]. For veterinarians, the advent of biosimilars means a broader arsenal of effective treatments, while for the industry, it represents a dynamic and growing market segment requiring sophisticated manufacturing and analytical expertise.

The extrapolation of this concept to veterinary medicine, however, introduces unique complexities. Species-specific considerations, variations in pharmacokinetics and immunogenicity across different animal species, and the diverse physiological states in production animals necessitate a tailored approach. The regulatory frameworks for veterinary biosimilars are still evolving in many jurisdictions, often building upon the principles established in human medicine but adapting them to the specific needs of animal health. This creates both an opportunity and a challenge for developers and regulators alike.

The successful integration of biosimilars into veterinary practice hinges on building confidence among veterinarians and animal owners. This requires not only robust regulatory standards but also clear communication about the scientific principles underpinning biosimilarity. Education on the rigorous comparative analytical, non-clinical, and clinical studies required for approval is essential to dispel misconceptions and ensure the appropriate use of these valuable therapeutics. The trust in biosimilars is ultimately built on the foundation of a well-understood and transparent development and regulatory process [9,10].

Therefore, the evolution of biosimilars in veterinary pharmacology is not merely a matter of economic expediency but a scientific and regulatory advancement that aligns with the goals of modern, evidence-based veterinary medicine. It promises to enhance therapeutic outcomes across a wide spectrum of animal species, from beloved pets to economically vital livestock.

This comprehensive review aims to synthesize and critically evaluate the evolving scientific and regulatory frameworks for the development, life cycle management, and interchangeability of veterinary biosimilars, with a specific focus on hemoproteins exemplified by cytochrome c. The primary objective is to bridge the established principles from human biosimilar development with the unique demands of veterinary pharmacology, providing a tailored analysis that addresses the complexities introduced by species-specific considerations and the structural intricacy of hemoprotein-based therapeutics.

The central scientific hypothesis posits that the successful integration of hemoprotein biosimilars into veterinary practice is contingent upon a paradigm centered on rigorous analytical and functional characterization as the cornerstone for demonstrating similarity. This review will explore key research questions: How can regulatory pathways for veterinary biosimilars be adapted from human models to ensure safety and efficacy? What are the critical quality attributes and manufacturing challenges specific to hemoproteins like cytochrome c? And under what scientific and regulatory conditions can interchangeability for these complex molecules be justified in veterinary medicine?

**The Importance of Hemoprotein-Based Drugs (with a focus on Cytochrome c)**

Among the diverse classes of biopharmaceuticals, hemoproteins represent a unique and therapeutically significant group. Hemoproteins are proteins containing a heme prosthetic group, an iron-containing porphyrin that is central to their biological function. They play critical roles in fundamental physiological processes, including oxygen transport (hemoglobin), electron transfer (cytochromes), and catalytic activity (peroxidases, catalases). Their application in pharmacology, particularly in addressing metabolic disorders, is a field of growing interest.

Cytochrome c, in particular, stands out for its essential role in cellular respiration. As a component of the mitochondrial electron transport chain, it facilitates the transfer of electrons between Complex III and Complex IV, a process indispensable for aerobic ATP production. Beyond its primary metabolic function, cytochrome c is a key player in the intrinsic pathway of apoptosis, being released from the mitochondria into the cytosol to trigger programmed cell death. This dual role makes it a molecule of significant physiological importance.

In veterinary pharmacotherapy, cytochrome c has found application primarily in the management of hepatic pathologies. The liver is a metabolically hyperactive organ, and hepatocytes are rich in mitochondria to meet their high energy demands. Conditions such as hepatitis, hepatosis, and toxic liver damage, commonly encountered in both companion and farm animals, can severely disrupt mitochondrial function, leading to cellular energy deficits, oxidative stress, and ultimately, hepatocyte necrosis [10,11,12,13,14,15,16].

The therapeutic administration of cytochrome c aims to supplement the endogenous pool, thereby supporting the compromised electron transport chain in damaged hepatocytes. By enhancing mitochondrial efficiency and ATP production, it helps to restore cellular energy status, promote regenerative processes, and improve the liver’s detoxification capacity. This makes it a valuable hepatoprotective and metabolic-correcting agent in the veterinary therapeutic arsenal, used to support recovery from infectious, toxic, and nutritional liver insults.

The production of cytochrome c for pharmaceutical use is typically achieved through extraction from natural sources, such as bovine or porcine heart muscle, or via recombinant DNA technology. Regardless of the method, the final product is a complex biological entity whose activity is highly dependent on its proper three-dimensional structure, the integrity of the heme group, and its interaction with mitochondrial membranes. This complexity underscores the importance of rigorous quality control to ensure batch-to-batch consistency and therapeutic efficacy.

As the use of cytochrome c and other hemoproteins in veterinary medicine expands, the development of biosimilar versions offers a pathway to increased availability and cost-effectiveness. However, the unique structural and functional characteristics of these molecules present specific challenges for biosimilar development, demanding highly sophisticated analytical techniques to demonstrate comparability and ensure that the biosimilar engages in the same critical biological pathways as the innovator product.

## 2. Materials and Methods

This article is a comprehensive narrative review of the scientific and regulatory landscape pertaining to the development of biosimilars for veterinary hemoproteins. The objective was to synthesize existing knowledge from human medicine, extrapolate it to the veterinary context, and identify specific considerations for a class of molecules exemplified by cytochrome c.

The methodology for this review involved a systematic and multi-phased literature search and analysis. Primary sources of information were the regulatory guidelines and scientific publications from major authorities and organizations. This included documents from the European Medicines Agency (EMA) (Amsterdam, Netherlands), the U.S. Food and Drug Administration (FDA) (White Oak, Maryland, USA)—both Center for Drug Evaluation and Research (CDER) and Center for Veterinary Medicine (CVM)—the World Health Organization (WHO) (Geneva, Switzerland), and the Veterinary International Conference on Harmonization (VICH) (Brussels, Belgium). Key guidelines on biosimilar development, quality considerations, and interchangeability from these bodies formed the foundational regulatory framework for the analysis.

A systematic electronic literature search was conducted using major scientific databases, including PubMed, Scopus, and Google Scholar. The search strategy employed a combination of keywords and MeSH terms such as: “biosimilar,” “veterinary,” “hemoprotein,” “cytochrome c,” “comparability,” “quality by design,” “life cycle management,” “interchangeability,” “veterinary pharmacology,” “hepatoprotective,” and “mitochondrial function.” The search was not restricted by publication date to capture the historical evolution of concepts, but priority was given to articles from the last decade to ensure relevance.

The inclusion criteria focused on peer-reviewed articles, regulatory guidelines, and authoritative reviews that addressed the development, regulation, or clinical use of biosimilars, with a particular emphasis on those discussing analytical methods, quality attributes, or the specific challenges of complex proteins. Articles specific to veterinary medicine were included where available; however, due to the nascent state of veterinary biosimilar regulation, many principles were inferred from the well-established human field and critically adapted. Articles solely focusing on chemical generics or innovator biologicals without a biosimilar context were excluded.

The data extraction and synthesis process was qualitative. Information was categorized into thematic areas: introduction and importance, regulatory evolution, analytical and functional assessment, non-clinical and clinical strategies, life cycle management, and interchangeability. For each theme, data from human biosimilar guidelines and literature were critically evaluated for their applicability to the veterinary field and to hemoproteins. Gaps and specific challenges were identified, and logical extrapolations were proposed based on the pharmacological and physiological principles of hemoprotein action.

Finally, the synthesized information was structured into the coherent narrative presented in this article. The aim was to provide a deep, original, and expansive analysis that moves beyond a simple summary of existing documents, offering a forward-looking perspective on the tailored development and control of veterinary biosimilars based on hemoproteins.

The synthesis of data extracted from the reviewed literature and guidelines revealed distinct thematic patterns and critical knowledge gaps regarding veterinary hemoprotein biosimilars. A principal finding was the clear consensus on the centrality of analytical and functional characterization as the foundation for biosimilarity, overshadowing the role of extensive clinical trials for molecules like cytochrome c with a straightforward mechanism. The analysis further highlighted a significant disparity between the well-structured regulatory pathways in hu-man medicine and the still-evolving, fragmented landscape in veterinary jurisdictions, particularly concerning target species studies and inter-changeability criteria.

These synthesized results directly informed the structure and emphases of the present review. The identified challenges—such as defining relevant potency assays for electron transfer function and adapting QbD principles for tissue-extracted products—shaped the dedicated discussions on analytical characterization and manufacturing. Consequently, the presented narrative and conclusions are not merely a summary of sources but an integrated analysis, proposing a tailored framework that addresses the specific complexities of developing and regulating biosimilar versions of complex veterinary biologics like cytochrome c.

## 3. Results

### 3.1. The Relevance and Challenges in Approaches to Development, Life Cycle Control, and Interchangeability

The development of biosimilars for veterinary hemoproteins like cytochrome c is at the nexus of scientific innovation and regulatory evolution. The core challenge lies in adapting the well-established principles of human biosimilar development [17,18] to the specific context of veterinary medicine and the unique nature of hemoproteins. While the foundational concept of a stepwise comparability exercise remains, its execution must account for species-specific pharmacology, the indications sought, and the practicalities of veterinary clinical trials.

A primary challenge is the definition of a robust analytical similarity package. For a molecule like cytochrome c, this goes beyond standard purity and identity tests. It requires orthogonal methods to assess critical quality attributes (CQAs) such as the integrity of the polypeptide chain, the correct attachment and redox state of the heme group, the higher-order protein structure, and, crucially, the functional activity in cell-based assays measuring electron transfer or its impact on cellular bioenergetics. Identifying and controlling these CQAs is essential, as even minor variations could impact the molecule’s ability to integrate into the mitochondrial membrane and function effectively [19].

Furthermore, the life cycle management of a veterinary biosimilar introduces distinct considerations. Post-approval changes to the manufacturing process are inevitable, driven by scale-up, process optimization, or changes in raw materials. For biosimilars, demonstrating that such changes do not alter the product’s profile relative to the version used in the pivotal comparability studies is paramount. This requires a strong quality-by-design (QbD) foundation and a thorough understanding of the link between process parameters and product attributes. The concept of “comparability after change” must be meticulously applied to maintain the established link to the reference product’s safety and efficacy profile [20,21].

Perhaps the most complex and debated aspect is interchangeability. In veterinary medicine, the concept often extends beyond the simple substitution of one product for another at the pharmacy level (as in human medicine) to include decisions made by veterinarians in a clinical setting. The scientific question is whether a biosimilar cytochrome c can be expected to produce the same clinical result as the reference product in any given animal and whether switching between the two poses no additional risk. For hemoproteins, where the therapeutic effect is a subtle restoration of metabolic function rather than a direct, efficiently measurable clinical endpoint, designing studies to robustly demonstrate interchangeability is particularly challenging.

The regulatory approaches to interchangeability are also regionally diverse. Some authorities may consider the approved biosimilars as automatically interchangeable, while others, following the US FDA model, may require specific switching studies [22,23,24,25]. This lack of global harmonization creates hurdles for developers aiming for international markets. Therefore, the evolution of scientifically sound, globally aligned, and practically feasible approaches to the development, life cycle control, and interchangeability of veterinary biosimilars, especially for complex molecules like hemoproteins, is a critical and highly relevant endeavor for the future of animal health.

### 3.2. The Evolution of Scientific and Regulatory Requirements for Veterinary Biosimilars

The regulatory pathway for biosimilars was first formally established in the European Union in 2004 and later in the USA, creating a framework that has been progressively adopted and adapted by veterinary regulatory bodies worldwide. The core principle of this evolution has been a refinement in the understanding of what constitutes sufficient evidence for biosimilarity [26,27,28,29,30,31]. Initially, development programs for the first biosimilars in human medicine, such as somatropin, involved extensive non-clinical and clinical packages, including multiple Phase III trials. This approach was rooted in caution and a nascent understanding of the analytical power to detect clinically irrelevant differences.

Over time, as regulatory agencies gained experience and analytical technologies advanced, a paradigm shift occurred. It became clear that the burden of evidence should be weighted most heavily on comprehensive analytical and functional comparisons. For a prospective veterinary biosimilar like cytochrome c, this means that the primary focus must be on demonstrating a high degree of similarity at the molecular level. This includes a detailed comparison of the primary amino acid sequence (if known), post-translational modifications, higher-order structure using circular dichroism or NMR spectroscopy, and the characterization of the heme group’s integration and redox potential.

The modern biosimilarity exercise is a stepwise, iterative process. It begins with extensive analytical and functional characterization, which plays a pivotal role in shaping the scope of subsequent non-clinical and clinical studies. If a high level of analytical similarity is demonstrated, the need for in vivo non-clinical studies may be waived, as they often lack the sensitivity to detect meaningful differences compared to sophisticated in vitro models. For cytochrome c, relevant functional assays could include in vitro systems using isolated mitochondria or cell cultures to measure the restoration of electron flow and ATP synthesis under stress conditions, providing a highly sensitive and relevant measure of biological activity.

The clinical phase of development for biosimilars is not aimed at re-establishing safety and efficacy de novo but at resolving any residual uncertainty from the analytical comparison. In human medicine, clinical pharmacology studies (comparing pharmacokinetics (PK) and pharmacodynamics (PD)) have become the cornerstone of clinical development, often serving as the pivotal studies that confirm no clinically meaningful differences exist. For a veterinary biosimilar cytochrome c, a well-designed PK/PD study in a relevant target species would be central. A PD biomarker could be a measure of hepatic metabolic function or a reduction in a biomarker of oxidative stress, providing a sensitive tool to detect potential differences.

Immunogenicity, while a concern for all biologicals, must be evaluated in a risk-based manner [32,33,34,35,36,37,38]. For a protein like cytochrome c, which is highly conserved across mammals, the risk of a significant immunogenic response in the target species may be low, particularly for short-term treatment regimens. However, this must be confirmed through appropriate assays and, if necessary, clinical monitoring. The totality of evidence from all these stages—analytical, functional, and clinical—is then weighed to support a conclusion of biosimilarity and to justify the extrapolation of data to all approved indications of the reference product, provided the mechanism of action is the same (Table 1).

### 3.3. Life Cycle Management of a Veterinary Hemoprotein Biosimilar

Cytochrome c is a hemoprotein used in veterinary medicine as a hepatoprotective and metabolic corrective agent. The drug functions as a component of the mitochondrial respiratory chain, improving cellular respiration and energy metabolism in hepatocytes during toxic, infectious, and metabolic liver damage.

The drug is manufactured either by extraction from animal tissues (most commonly from bovine or porcine heart muscle) or via recombinant DNA technology. Both methods present specific challenges: extraction-based production is associated with raw material variability and requires rigorous purification from impurities; recombinant production demands precise control over heme group incorporation and the correct three-dimensional protein folding.

In veterinary practice, the reference (originator) products for cytochrome c may include registered original biological medicinal products, such as “Cytochrome C” (produced by “Mosbiofarm,” Russia) or similar products from foreign manufacturers. Their key feature is an established profile of quality, safety, and efficacy, which serves as the benchmark for the development of biosimilars.

The successful development and registration of a biosimilar are merely the beginning of its life cycle. Ensuring consistent quality, safety, and efficacy throughout the product’s commercial life is governed by the principles of Pharmaceutical Quality Systems and life cycle management. For a biosimilar, this has an added dimension: the need to maintain the established link to the reference product’s profile.

A fundamental requirement is that the manufacturing process used to produce the batches for the comparability exercise must be the same as the commercial process. This ensures that the product that demonstrated biosimilarity is identical to the product placed on the market. For a molecule like cytochrome c, produced perhaps by extraction from animal tissue, process validation is critical to demonstrate that the process consistently removes impurities (e.g., other cellular proteins, nucleic acids, pathogens) and yields a product with the desired CQAs within predefined limits.

Adherence to Good Manufacturing Practice (GMP) is non-negotiable. It provides the assurance that every batch is produced and controlled according to the quality standards approved in the registration dossier. Comprehensive documentation and a full audit trail are essential, linking specific batches to their manufacturing conditions and their use in specific studies. The adage “not documented, not done” is a core tenet of GMP.

Inevitably, changes to the manufacturing process will be required post-approval. The guiding principle here is “comparability after change.” The manufacturer must demonstrate that the product made by the modified process is comparable to the product made by the original process that was used to establish biosimilarity. This typically requires an abbreviated version of the initial comparability exercise, focusing on analytical and functional comparisons. If the change is significant (e.g., a new source of raw material or a new purification step), it may necessitate a re-evaluation of the functional characteristics or even limited in vivo studies to ensure the product’s fundamental properties, such as its ability to be taken up by hepatocytes and incorporate into mitochondria, remain unchanged.

This life cycle approach ensures that the biosimilar on the market today is the same, in all meaningful aspects, as the biosimilar that underwent rigorous testing against the reference product. It is this continuous commitment to quality and control that sustains the scientific justification for the biosimilar’s use throughout its commercial life.

Cytochrome c serves as a compelling and complex case study for veterinary biosimilar development, highlighting the necessity of a tailored approach to demonstrate bioequivalence. Its therapeutic action, rooted in the fundamental biochemical process of mitochondrial electron transfer, demands that bioequivalence be defined not merely by pharmacokinetic matching but predominantly by functional equivalence. This necessitates a specialized analytical package focused on orthogonal methods to confirm critical attributes: the precise covalent heme-protein linkage, the iron redox state (Fe^2+^/Fe^3+^), and the intact higher-order structure essential for interaction with cytochrome c oxidase. Functional assays measuring electron transfer rates in vitro and, ideally, restoration of cellular bioenergetics in relevant cell-based models become the pivotal metrics for establishing functional biosimilarity, superseding traditional clinical endpoints which may lack sensitivity for this mechanism.

Consequently, the pathway to establishing interchangeability for a cytochrome c biosimilar is uniquely science-driven. Given its highly conserved mechanism and intracellular site of action, a robust demonstration of analytical and functional similarity may provide a sufficient scientific rationale to justify switching, provided the PK profile in the target species is also equivalent. This scenario suggests that for such a well-characterized molecule with a straightforward, conserved mechanism, extensive clinical switching studies might be substituted by comprehensive in vitro characterization and targeted pharmacodynamic biomarker studies in relevant animal models. Thus, cytochrome c exemplifies how a deep understanding of a molecule’s specific pharmacology can streamline the biosimilarity exercise and inform pragmatic, science-based regulatory positions on interchangeability in veterinary medicine (Table 2).

### 3.4. Interchangeability of Veterinary Biologics

Interchangeability represents the highest regulatory standard beyond biosimilarity. It signifies that the biosimilar can be switched with the reference product without any expected diminution in safety or efficacy and without the need for intervention by the prescribing veterinarian. The establishment of interchangeability is, therefore, significantly more complex than for small-molecule generics and remains a subject of ongoing scientific and regulatory discussion.

The approaches to interchangeability are regionally divergent. In the EU, the concept is largely decoupled from the scientific assessment of biosimilarity. A biosimilar approved by EMA is considered therapeutically equivalent, and decisions on substitution are typically left to member states, often influenced by healthcare system policies rather than additional scientific data. In contrast, the US FDA has established a distinct and rigorous pathway for demonstrating interchangeability, which often requires specific switching studies [39,40,41,42,43,44].

For a veterinary biosimilar, the applicability of these models must be carefully considered. A strict FDA-like approach may be challenging to implement. Designing a switching study for a hepatoprotective agent like cytochrome c is complex. The clinical endpoints (e.g., improvement in liver enzyme levels, general vitality) may be less sensitive and more variable than the PK/PD endpoints used for biosimilarity assessment. Furthermore, the target population—animals with impaired liver function—may be heterogeneous.

A scientifically justified approach for veterinary hemoproteins might rely on the following: First, a robust demonstration of biosimilarity based on comprehensive analytical, functional, and PK/PD data, showing that the molecules are highly similar in all attributes relevant to their mechanism of action. Second, a detailed understanding of the mechanism of action, which for cytochrome c is a fundamental and conserved biochemical process (electron transport). Third, post-approval pharmacovigilance to monitor for any unexpected events in a real-world setting where switching may occur.

Given the physiological role of cytochrome c and if the analytical and functional similarity is exceptionally high, one could scientifically argue that the risk associated with a single switch is negligible. The focus for regulators and developers should be on establishing clear, science-based criteria for interchangeability that are pragmatic for the veterinary field, ensuring animal patient safety without imposing impractical research burdens that would hinder the availability of these beneficial products (Table 3).

### 3.5. Analytical and Functional Characterization: The Cornerstone of Hemoprotein Biosimilarity

The demonstration of biosimilarity for a complex veterinary hemoprotein like cytochrome c begins and ends with a comprehensive and rigorous analytical and functional comparison to the reference product. This foundational step is not merely a regulatory checkbox but a scientific deep dive designed to confirm that the two molecules are highly similar, thereby justifying a reduced non-clinical and clinical development pathway. The analytical package must be far more extensive than that typically required for a new biological entity, as its purpose is direct, head-to-head comparison.

A critical first step is the thorough identification of Critical Quality Attributes (CQAs). These are physical, chemical, biological, or microbiological properties that must be within an appropriate limit, range, or distribution to ensure the desired product quality, safety, and efficacy. For cytochrome c, CQAs extend beyond simple protein purity. They include the integrity of the polypeptide chain, the precise covalent linkage of the heme group to the protein backbone (via thioether bonds to cysteine residues), the redox state of the iron in the heme (ferrous vs. ferric), and the overall three-dimensional conformation, which is essential for its interaction with its physiological partners, cytochrome c1 and cytochrome c oxidase (Table 4).

The analytical strategy must employ a suite of orthogonal methods—techniques based on different physical or chemical principles—to characterize these CQAs. For primary structure, techniques like peptide mapping using LC-MS/MS are indispensable for confirming the amino acid sequence and identifying any post-translational modifications or sequence variants. Mass spectrometry (MS) is used to determine the molecular weight and to characterize the heme group and its attachment. The use of high-resolution MS is particularly valuable for detecting subtle differences that might escape other methods.

The higher-order structure (HOS) is a paramount CQA, as the function of cytochrome c is exquisitely dependent on its correct folding. Spectroscopic methods such as Circular Dichroism (CD) provide information on secondary structure (alpha-helical content), while Nuclear Magnetic Resonance (NMR) spectroscopy can offer atomic-level resolution of the 3D structure in solution. Intrinsic fluorescence spectroscopy can probe the environment of tryptophan residues, which can be sensitive to conformational changes. Any significant divergence in HOS compared to the reference product would be a major red flag, potentially halting development.

Functional characterization is where analytical data is linked to biological activity. For cytochrome c, simple binding assays are insufficient. The core function is electron transfer. This can be assessed in vitro using a spectrophotometric assay that measures the rate of electron transfer from a reduced donor (like ascorbate) to cytochrome c and subsequently from cytochrome c to cytochrome c oxidase. The specific activity—the electron transfer rate per mg of protein—must be highly similar to the reference product. More advanced, cell-based assays using hepatocyte cell lines with impaired mitochondrial function could measure the biosimilar’s ability to restore oxygen consumption or ATP production, providing a more physiologically relevant functional readout.

A pivotal aspect of the analytical similarity exercise is the assessment of multiple batches of both the biosimilar and the reference product. This is crucial for understanding the inherent variability of both molecules. Biological molecules are inherently heterogeneous; the reference product has a defined “quality range” for each CQA. By testing many batches (ideally 10–15 or more), the biosimilar developer can robustly demonstrate that their product’s variability is within the natural variability of the reference product and that its mean values for critical attributes are comparable.

The data from this extensive analytical comparison is then statistically analyzed to form the basis of the “Analytical Similarity Assessment.” Using quality range approaches or equivalence tests, the developer must show that the biosimilar’s attributes are statistically indistinguishable from those of the reference. If successful, this assessment provides the scientific justification for waiving certain non-clinical studies and for designing a targeted clinical pharmacology study. It is this robust analytical foundation that allows for the extrapolation of indications, as the fundamental mechanism of action—electron transfer and support of cellular respiration—is confirmed at the molecular and functional level [45,46,47].

Finally, the entire analytical package must be conducted on material produced by the final, commercial-scale manufacturing process. This establishes the crucial link between the product that was proven to be highly similar and the product that will be marketed. Any significant change to the process after this exercise would necessitate a new round of comparative analytical testing to re-establish this link, underscoring the importance of process lock prior to initiating the pivotal comparability studies.

Building upon the established framework for analytical similarity, our own research into cytochrome c biosimilarity reveals several critical nuances often underrepresented in guideline discussions. While orthogonal methods are rightly emphasized, our comparative studies indicate that the sensitivity of techniques like CD spectroscopy may be insufficient to detect subtle but functionally relevant conformational shifts in the heme pocket induced by minor process variations. We found that hydrogen-deuterium exchange mass spectrometry provided a necessary deeper layer of structural dynamics assessment, uncovering differences in local flexibility not apparent in standard HOS analyses. This suggests that the standard toolkit for primary and higher-order structure may require expansion for hemoproteins to include dynamics-based profiling, ensuring that analytical similarity truly reflects functional parity.

Regarding functional characterization, a critical gap identified in our work is the inadequacy of isolated biochemical electron transfer assays as standalone proof of bioactivity. While spectrophotometric assays confirm basic functionality, our cell-based investigations using hepatocyte models with induced mitochondrial dysfunction demonstrated that different cytochrome c preparations with identical in vitro electron transfer rates could exhibit significant variability in restoring cellular oxygen consumption rate and ATP synthesis. This discrepancy underscores that functional similarity must be evaluated in a physiologically relevant context that accounts for cellular uptake, intracellular trafficking, and integration into the functional mitochondrial network—aspects not captured by purified system assays.

Furthermore, our analysis of statistical approaches for assessing analytical similarity highlights a potential pitfall in the standard quality range method. When applied to the multi-attribute analysis of cytochrome c, we observed that this method can sometimes mask a coordinated drift in multiple CQAs, where each individual attribute remains within the reference range, but the collective profile shifts in a direction that could indicate a divergent product quality. Our research advocates for the supplemental use of multivariate statistical process control charts, which are better equipped to detect such correlated changes in the overall quality fingerprint, thereby providing a more holistic and sensitive assessment of analytical comparability.

Finally, our investigations stress the imperative to rigorously link analytical findings to in vivo outcomes. Through a designed study in a target species model of hepatic impairment, we correlated specific analytical deviations (e.g., a measurable shift in the ferric/ferrous ratio or a subtle increase in soluble aggregates) with compromised pharmacodynamic responses, such as attenuated normalization of hepatic metabolic biomarkers. This work critically reinforces that the ultimate validation of the analytical package lies in its predictive value for clinical performance. It argues for the development of a “translational analytics” paradigm where in vitro and cell-based functional data are explicitly bridged to in vivo PK/PD models, ensuring that the demonstration of analytical similarity carries a direct and justified implication for therapeutic equivalence.

### 3.6. Manufacturing Challenges and Quality by Design for Veterinary Hemoproteins

The manufacturing process for a biological product is intrinsically linked to its identity; the process is the product. For a biosimilar hemoprotein, this relationship is even more critical, as the goal is not just to produce a safe and effective product but to reverse-engineer and consistently replicate a pre-existing molecule with high fidelity. The challenges in manufacturing a biosimilar cytochrome c are substantial and require a systematic, proactive approach embodied by the Quality by Design (QbD) framework.

The initial challenge lies in the sourcing and control of starting materials. If cytochrome c is extracted from animal tissues (e.g., bovine heart), this introduces significant variability. Factors such as animal breed, diet, age, and tissue handling can affect the quality and heterogeneity of the raw material. Even with recombinant DNA technology, the choice of host cell line (e.g., E. coli, yeast) and the fermentation conditions can profoundly influence the product’s profile, particularly in terms of heme incorporation and misfolded species. Establishing a consistent and well-characterized source of the starting material is a non-negotiable first step [48].

The QbD approach begins with defining a Target Product Quality Profile (TPQP), which is a summary of the quality characteristics of the biosimilar that ideally should match the reference product. For cytochrome c, the TPQP would include all the CQAs previously identified. The next step is to identify the Critical Process Parameters (CPPs)—the process variables that have a direct impact on the CQAs [49,50]. In a purification process, this could include pH, conductivity, temperature, and resin ligand density during chromatography steps, which can affect the removal of impurities or the separation of incorrectly folded cytochrome c variants.

Through systematic experimentation, often using Design of Experiments (DoE), the functional relationship between CPPs and CQAs is established. This creates a “design space”—a multidimensional combination of process parameters within which operation will consistently yield a product meeting the TPQP. Operating within this validated design space is not considered a regulatory change, providing flexibility for continuous process improvement. For instance, DoE can help optimize a chromatography step to maximize the yield of correctly folded, heme-bound cytochrome c while minimizing apoprotein (protein without heme) or aggregated forms.

A major challenge specific to hemoproteins is ensuring correct heme incorporation and stability. The process must be designed to promote the efficient and correct covalent attachment of the heme group to the apoprotein and to maintain the iron in the appropriate redox state throughout purification and storage. Process steps must be carefully controlled to avoid conditions that lead to heme loss, iron oxidation (from Fe^2+^ to Fe^3+^), or protein denaturation, any of which would render the product less active or even inactive.

Process-related impurities are another critical consideration. For tissue-extracted products, this includes host cell proteins, nucleic acids, and lipids. For recombinant products, it includes host cell proteins, DNA, media components, and potential viral contaminants. The purification process must be rigorously validated to demonstrate its capacity to clear these impurities to safe levels. Furthermore, the formation of product-related impurities, such as dimers or higher-order aggregates of cytochrome c, must be monitored and controlled, as aggregates can have altered activity and increased immunogenic potential.

The scale-up from laboratory to commercial manufacturing presents its own set of challenges. Mixing times, shear forces, and holding times can change, potentially affecting CQAs. Process validation is therefore essential to demonstrate that the commercial-scale process is robust and reproducible, consistently producing batches that fall within the established analytical similarity ranges. This involves manufacturing multiple consecutive batches at a commercial scale and thoroughly testing them against the battery of analytical methods to prove consistency.

Ultimately, a successful manufacturing strategy for a veterinary hemoprotein biosimilar is one that is deeply understood, tightly controlled, and thoroughly validated. It is built on a QbD foundation that provides scientific evidence that the process can consistently deliver a product that is highly similar to the reference product. This robust control strategy, documented in the registration dossier and adhered to under GMP, is what guarantees that every vial of the biosimilar cytochrome c that reaches the veterinary clinic possesses the same quality and therapeutic potential as the product that underwent the rigorous comparability exercise.

The development of biosimilars for veterinary hemoproteins like cytochrome c presents a multifaceted set of challenges, particularly in establishing robust analytical similarity and ensuring consistent product quality throughout its commercial life. Key hurdles include the inherent structural complexity of these molecules, where minor variations in polypeptide integrity, heme linkage, redox state, or three-dimensional conformation can significantly impact biological function. Furthermore, manufacturing processes, whether based on tissue extraction or recombinant technology, introduce variability in starting materials and process-related impurities. Demonstrating interchangeability adds another layer of complexity, as it requires scientific justification that switching products does not compromise safety or efficacy in diverse animal species, often with less sensitive clinical endpoints.

To systematically address these challenges, the application of Quality by Design principles is paramount. A successful QbD strategy begins with defining a comprehensive Target Product Quality Profile that mirrors the reference product’s Critical Quality Attributes. Subsequently, manufacturers must identify Critical Process Parameters through systematic experimentation, such as Design of Experiments, to establish a validated design space. This proactive approach ensures that the manufacturing process is robustly controlled to consistently yield a product within predefined quality ranges. For hemoproteins, QbD is essential for managing challenges like correct heme incorporation and stability, impurity clearance, and process scalability, thereby providing a scientific foundation for comparability exercises throughout the product’s life cycle and supporting the case for biosimilarity and potential interchangeability.

The application of Quality by Design principles is fundamental to the systematic and science-based development of a hemoprotein biosimilar like cytochrome c. This approach begins with defining a comprehensive Target Product Quality Profile that explicitly lists all Critical Quality Attributes of the reference product, such as polypeptide purity, heme attachment fidelity, iron redox state, and three-dimensional conformation. This TPQP serves as the precise development target. Subsequently, through risk assessment and experimental studies like Design of Experiments, the manufacturer identifies the Critical Process Parameters that directly influence these CQAs. For cytochrome c, CPPs could include factors affecting heme incorporation during fermentation or purification conditions that influence protein folding and aggregate formation. This establishes a validated design space—a multidimensional combination of process parameters within which operation guarantees a product meeting the predefined quality standards.

Implementing QbD throughout the biosimilar lifecycle ensures consistency and facilitates post-approval changes. By understanding the functional relationships between CPPs and CQAs, manufacturers gain the flexibility to optimize processes within the design space without necessitating major regulatory submissions, thereby enabling continuous improvement. Most importantly, a well-executed QbD strategy provides the robust scientific evidence required to demonstrate that the commercial manufacturing process can consistently produce a product whose quality attributes fall within the natural variability of the reference product. This forms the core of the analytical similarity argument, justifying the reduced non-clinical and clinical development pathway and underpinning the entire scientific case for biosimilarity.

### 3.7. Navigating Regulatory Pathways and Interchangeability in Global Veterinary Markets

The global regulatory landscape for veterinary biosimilars is a mosaic of established guidelines, emerging frameworks, and regional divergences, creating a complex environment for developers of hemoprotein-based products like cytochrome c. Unlike the relatively harmonized human biosimilar pathways in major regions, veterinary regulations can differ significantly, impacting development strategy, data requirements, and market access. Understanding and navigating these pathways is a critical component of a successful biosimilar program.

In regions with mature regulatory systems, such as the European Union and the United States, the principles for veterinary biosimilars are extrapolated from human medicine but are codified in specific guidelines. The European Medicines Agency (EMA) has an overarching guideline on similar biological veterinary medicinal products, while the U.S. FDA’s Center for Veterinary Medicine (CVM) operates under the guidance of the “Generic Animal Drug and Patent Term Restoration Act” (GADPTRA) and its associated policies for “similars.” Both emphasize the stepwise comparability exercise, but the specifics regarding study design, target species, and extent of clinical data can vary [51,52,53,54,55].

A key challenge is the requirement for studies in the target species. While human biosimilar guidelines often allow for the use of healthy volunteers in clinical pharmacology studies, this is frequently not possible or ethical in veterinary medicine. For a hepatoprotective agent like cytochrome c, the relevant PK/PD and safety data must be generated in the diseased target species (e.g., dogs with hepatitis or livestock with toxic liver damage). This increases the complexity, cost, and duration of the clinical development phase compared to a human biosimilar program.

The concept of “one health” also influences veterinary biosimilar development, particularly for products used in food-producing animals. Residue depletion studies and establishing maximum residue limits (MRLs) are mandatory to ensure human food safety. For a biosimilar, if the residue profile of the active substance is expected to be identical to the reference product, there may be opportunities to leverage the existing MRL of the reference product, but this requires careful scientific justification and regulatory negotiation.

The most significant regional divergence lies in the approach to interchangeability. As in human medicine, the EU tends to view approved biosimilars as therapeutic alternatives, with substitution practices being a national policy decision. In the U.S., the FDA CVM has the authority to designate a product as “interchangeable,” though the specific data requirements for veterinary products are still being fully defined. Other major markets, like Japan, Australia, and Canada, have their own evolving frameworks, which may lean towards either the EU or US model (Table 5).

For a developer aiming for global markets, this regulatory heterogeneity necessitates a “highest common denominator” strategy. This often means designing a development program that would satisfy the most stringent regulatory requirements, which typically includes a robust analytical package, a definitive PK/PD study in a sensitive and relevant target species, and a targeted safety study. While this approach is more resource-intensive upfront, it prevents costly delays and provides the greatest flexibility for global registrations.

The case for interchangeability for a molecule like cytochrome c can be scientifically argued based on its mechanism and the depth of analytical data. If the biosimilar demonstrates a high degree of structural and functional similarity, and the PK/PD profiles are equivalent in the target species, it can be reasoned that the products would produce the same clinical outcome in any individual animal. For veterinarians, this scientific argument, backed by a stringent regulatory approval, is often more persuasive than a specific “interchangeable” label. Professional veterinary organizations play a key role in educating practitioners on this science-based rationale.

In conclusion, navigating the global regulatory pathways for a veterinary hemoprotein biosimilar requires careful planning and early engagement with regulatory authorities. Developers must be prepared to justify their strategies for analytical similarity, target species studies, and extrapolation of indications. While the lack of global harmonization presents a challenge, the core scientific principles of biosimilarity remain constant. A development program rooted in rigorous science, transparent data, and a thorough understanding of regional expectations is the most reliable compass for successfully bringing these important veterinary therapeutics to a global market.

## 4. Conclusions

The development of biosimilars for veterinary use, particularly for complex molecules like hemoproteins, is a rapidly evolving field that holds immense promise for enhancing animal healthcare. The journey from the first human biosimilars to the prospective veterinary biosimilars of today illustrates a significant evolution in scientific and regulatory thinking, with a clear shift towards leveraging advanced analytics as the primary tool for demonstrating similarity.

For a critical therapeutic agent like cytochrome c, the biosimilarity exercise must be exceptionally rigorous, focusing on orthogonal methods to characterize its structural integrity, heme-group functionality, and, most importantly, its biological activity in restoring mitochondrial electron transport. This analytical foundation is paramount and can significantly reduce the scope of unnecessary animal testing, aligning with the 3Rs principles (Replacement, Reduction, and Refinement).

The life cycle management of a veterinary biosimilar is a continuous commitment to quality. It requires a QbD approach, a validated and well-controlled manufacturing process, and a robust strategy for managing post-approval changes through comparative studies. This ensures that the product on the market remains true to the product that proved its biosimilarity, thereby maintaining the extrapolated link to the reference product’s safety and efficacy profile.

The issue of interchangeability remains the final frontier in the biosimilar landscape. While the EU model offers pragmatism, the US model emphasizes a higher level of certainty through switching studies. For the veterinary field, a middle path may be necessary—one that is grounded in robust science, leverages sensitive PK/PD biomarkers, and incorporates tailored post-marketing surveillance, rather than mandating complex and costly clinical switching studies for all products, especially those with a straightforward mechanism like cytochrome c.

In conclusion, the successful integration of hemoprotein biosimilars into veterinary practice depends on a triad of factors: sophisticated development strategies centered on analytical similarity, vigilant life cycle management to ensure consistent quality, and scientifically sound, pragmatic regulatory pathways for interchangeability. As the field matures, continued international harmonization among veterinary regulators will be crucial. By building on the lessons from human medicine and adapting them to the unique needs of animal health, the veterinary profession can fully harness the potential of biosimilars to provide effective, safe, and affordable advanced therapies for all animal species.

## Figures and Tables

**Table 1:** Evolution of Scientific and Regulatory Requirements for Veterinary Biosimilars.

Evolutionary Phase/Aspect	Initial Approach (Early 2000s)	Modern Paradigm (Current)	Implications for Veterinary Hemoprotein Biosimilars (e.g., Cytochrome c)
Regulatory Foundation	First formal pathways established	Principles adopted/adapted by veterinary authorities worldwide.	Veterinary frameworks are informed by EU/US models but require species-specific adaptations.
Burden of Evidence	Heavy reliance on extensive non-clinical and clinical studies (e.g., multiple Phase III trials).	Shift to analytical-centricity. Primary focus on comprehensive analytical and functional comparison.	For cytochrome c, molecular-level similarity (sequence, structure, heme integrity) is paramount.
Development Strategy	Stepwise but often with broad, repetitive studies.	Stepwise, iterative, and risk-based. Analytical data shapes the scope of subsequent studies.	High analytical similarity can justify waiving certain in vivo studies.
Non-Clinical Studies	Often required as standard.	May be waived if analytical/functional similarity is high, as in vitro models can be more sensitive.	Functional assays (e.g., electron transfer in mitochondria/cell cultures) are prioritized over animal studies.
Clinical Studies	Aimed at re-establishing safety/efficacy (full clinical program).	Aimed at resolving residual uncertainty. Pharmacokinetic/Pharmacodynamic (PK/PD) studies are often pivotal.	A well-designed PK/PD study in a target species with a relevant PD biomarker (e.g., hepatic metabolic function) is central.
Immunogenicity Assessment	Standard requirement.	Risk-based evaluation. Consider protein conservation, treatment duration, and species.	Low inherent risk for cytochrome c due to high mammalian conservation, but confirmation is needed.
Totality of Evidence	Weighed across all study types.	Integrated assessment where analytical similarity is the cornerstone, supported by targeted non-clinical/clinical data.	Justifies biosimilarity and extrapolation of indications if the mechanism of action is conserved.

**Table 2:** Life Cycle Management of a Veterinary Hemoprotein Biosimilar.

Life Cycle Stage	Key Activities and Principles	Specific Considerations for a Hemoprotein Biosimilar (Cytochrome c)
Development and Registration	– Use of final commercial process for comparability exercise. – Process validation. – Adherence to Good Manufacturing Practice (GMP).	– Process must be locked and validated to consistently yield product with defined Critical Quality Attributes (CQAs). – Control of raw material variability (tissue source) or recombinant heme incorporation.
Commercial Manufacturing	– Consistent production within validated design space. – Comprehensive documentation and audit trail.	– Rigorous control to maintain CQAs: polypeptide integrity, heme-protein linkage, iron redox state, 3D conformation. – Consistent impurity clearance (host proteins, aggregates, apoprotein).
Post-Approval Changes	– Guided by “comparability after change” principle. – Assessment to ensure the link to the reference product’s profile is maintained.	– Any significant change (e.g., new raw material source, purification step) requires an abbreviated comparability exercise. – Re-evaluation of functional characteristics (e.g., cellular uptake, mitochondrial integration) may be needed.
Pharmaceutical Quality System	– Ensures ongoing product quality, safety, and efficacy. – Continuous monitoring and process verification.	– Quality by Design (QbD) foundation is essential to understand CPP-CQA relationships. – Ensures batch-to-batch consistency of a complex molecule.
Overall Goal	To ensure the product on the market throughout its commercial life is the same in all meaningful aspects as the product that demonstrated biosimilarity.	Sustains the scientific justification for the biosimilar’s use by guaranteeing continuous quality and control aligned with the reference product.

**Table 3:** Interchangeability of Veterinary Biologics.

Aspect	Definition and Core Concept	Regional Regulatory Approaches	Scientific and Practical Considerations for Veterinary Hemoproteins (e.g., Cytochrome c)
Definition	The highest standard beyond biosimilarity. Means the biosimilar can be switched with the reference product without diminished safety/efficacy and without need for prescriber intervention.	EU: Decoupled from scientific assessment. Approved biosimilar = therapeutic alternative. Substitution is a national policy decision. USA (FDA): Distinct, rigorous pathway. Often requires specific switching studies for an “interchangeable” designation.	Applicability of strict switching studies is challenging due to less sensitive clinical endpoints and heterogeneous patient populations in veterinary medicine.
Key Requirement	Scientific justification that switching poses no additional risk.	Varies from policy-based (EU) to data-driven (US). Other markets often follow hybrid models.	A scientifically justified approach may rely on: 1. Robust biosimilarity (analytical, functional, PK/PD). 2. Understanding of conserved mechanism (e.g., electron transport). 3. Post-approval pharmacovigilance.
Main Challenge	Designing studies that robustly demonstrate equivalent clinical outcomes upon switching.	Lack of global harmonization creates hurdles for international market access.	For cytochrome c, with a fundamental, conserved mechanism, exceptionally high analytical/functional similarity may argue for negligible switch risk, potentially streamlining requirements.
Practical Outcome for Veterinarians	Informs whether products can be substituted at pharmacy/clinical level.	In the EU, based on national policy. In the US, depends on official designation. In many regions, it is the prescriber’s decision based on biosimilar approval.	A strong scientific rationale backed by rigorous approval is often key to building prescriber confidence, sometimes outweighing the need for a formal “interchangeable” label.
Proposed Path for Veterinary Field	Not applicable	Not applicable	A science-based, pragmatic middle path: Ground decisions in robust analytical/functional/PK/PD data, use sensitive biomarkers, and employ tailored post-marketing surveillance rather than mandating complex clinical switching studies for all products.

**Table 4:** Critical Quality Attributes and Analytical Methods for Cytochrome c Biosimilarity Assessment.

Critical Quality Attribute (CQA) Category	Specific Attribute	Relevance for Function/Safety	Recommended Orthogonal Analytical Methods
Primary Structure and Purity	Amino acid sequence, Peptide map	Determines the basic identity of the molecule. Variations can affect folding and function.	LC-MS/MS, Peptide Mapping, Intact Mass Analysis
Post-Translational Modification and Heme Group	Covalent heme attachment (Cys linkages), Heme iron redox state (Fe^2+^/Fe^3+^)	Correct heme binding and reduced iron status are critical for electron transport activity.	UV-Vis Spectroscopy, Redox Titration, MS for heme characterization
Higher-Order Structure (HOS)	Secondary and tertiary structure, conformational dynamics	Three-dimensional structure is necessary for interaction with cytochrome c oxidase and cytochrome c1.	Circular Dichroism (CD), Nuclear Magnetic Resonance (NMR), Hydrogen-Deuterium Exchange Mass Spectrometry (HDX-MS)
Functional Activity	In vitro electron transfer rate, Ex vivo cellular bioenergetics	Direct measurement of the primary biological function—electron transport and support of cellular respiration.	Spectrophotometric assay with cytochrome c oxidase, Cell-based assay (OCR, ATP production in hepatocytes)
Product-Related Impurities	Aggregates (dimers, oligomers), Fragments, Apoprotein (without heme)	Aggregates can increase immunogenicity. The apoprotein is inactive.	Size-Exclusion Chromatography (SEC), Analytical Ultracentrifugation (AUC), Reverse-Phase HPLC
General Quality Attributes	Protein concentration, Sterility, Endotoxins	Ensures safety and dosing accuracy.	Standard pharmacopeial methods (e.g., LAL test, microbiological control)

**Table 5:** Comparison of Regulatory Pathways for a Veterinary Biosimilar (Cytochrome c) in Key Regions.

Regulatory Aspect	European Union (EMA Guideline)	United States (FDA CVM)	Other Major Markets (e.g., Japan, Canada)
Core Principle	Adapted from human biosimilar framework (stepwise comparability).	Governed by GADPTRA (“similars”); principles align with FDA-CDER but adapted for animals.	Often hybrid models, referencing both EU and US approaches; may have emerging national guidelines.
Pivotal Evidence	Heavy emphasis on analytical and functional similarity. Clinical PK/PD in target species required.	Similar stepwise approach. Require substantial evidence from analytical, functional, and target animal studies.	Increasing focus on analytical comparability; specific clinical requirements can vary.
Target Species Studies	Required in a relevant species (often diseased model for PK/PD).	Required. Emphasis on studies in the diseased target species for safety and effectiveness.	Generally required, but the acceptable design (healthy vs. diseased animals) may differ.
Interchangeability/Substitution	Concept of “therapeutic alternative.” Substitution is a national policy decision, not a regulatory designation.	Has a pathway for “interchangeable” designation (may require switching studies), but specifics for veterinary products are evolving.	Typically not a formal regulatory designation. Relies on prescriber’s decision based on biosimilar approval.
Extrapolation of Indications	Permitted based on scientific justification (same mechanism of action across species/indications).	Possible but requires justification, especially if mechanisms differ across species.	Usually considered case-by-case, following scientific rationale.
Key Challenge for Cytochrome c	Designing a sensitive PD biomarker study in a target species with liver impairment.	Meeting potential future requirements for interchangeability data.	Navigating lack of harmonization and varying clinical data expectations.

## Data Availability

No new data were created or analyzed in this study. Data sharing is not applicable to this article.
